# Mechanisms of Quality Preservation in Golden Pomfret Fish Balls Treated with Ultra-High Pressure During Freeze–Thaw Cycles

**DOI:** 10.3390/foods14193342

**Published:** 2025-09-26

**Authors:** Jiawen Liu, Xinyao Zeng, Jiaqi Zhao, Yunfeng Chi, Lin Xiu, Mingzhu Zheng, Huimin Liu

**Affiliations:** 1 College of Food Science and Engineering, Jilin Agricultural University, Changchun 130118, China; 2 National Engineering Laboratory for Wheat and Corn Deep Processing, Changchun 130118, China

**Keywords:** ultra-high pressure, golden pomfret fish balls, freeze–thaw cycle, textural characteristics, protein oxidation

## Abstract

The rising demand for convenient, nutritious foods necessitates improved freeze–thaw (F-T) stability in frozen fish balls; however, traditional thermal processing fails to prevent moisture loss, textural degradation, and oxidation. Therefore, this study systematically investigated the effect of ultra-high pressure (UHP) treatment on the quality of golden pomfret fish balls (*Trachinotus ovatus*) using two-step heating as a control during the F-T cycles. The results showed that compared to two-step heating, UHP significantly reduced the thawing loss (0.68 times) and centrifugal water loss (2.43 times) by enhancing the water-binding capacity (15–20%) and forming denser gel networks. Microstructural analysis revealed that UHP resulted in a more compact internal structure, reduced porosity, altered ice-crystal geometry, and a slower recrystallization rate of the fish balls. Furthermore, UHP effectively reduced protein oxidation (34.53% lower carbonyl increase) and lipid peroxidation (15.6% lower TBARS value) after five F-T cycles compared to the control. Correlation analysis confirmed the dual role of UHP in the regulation of oxidative and structural stability. These findings provide a new technological approach for processing and storing fish balls.

## 1. Introduction

Driven by increasing consumer demand for convenience, nutrition, and high-quality protein sources, the global seafood market based on surimi is projected to exceed 1.6 billion in value by 2030 and will continue to expand rapidly. Golden pomfret (*Trachinotus ovatus*) fish balls have garnered significant attention because of their unique texture and nutritional value, particularly as a rich source of omega-3 fatty acids and high-quality proteins. Frozen storage is the primary method used to preserve fish balls [1]. However, temperature fluctuations may occur during the manufacturing, storage, transport, and distribution of surimi products. During multiple freeze–thaw (F-T) cycles, the quality of fish balls deteriorates, affecting their flavor and color and reducing their economic value. Traditional processing methods, such as heat treatment and chemical additives, often fail to effectively address food preservation challenges while preserving the nutritional properties and sensory quality, thereby underscoring the urgent need for innovative preservation technologies.

Ultra-high pressure (UHP) technology is a cold process that is a purely physical technique widely used for fish processing and storage. It effectively inactivates microbes and modulates enzyme activity, while preserving heat-sensitive nutrients and bioactive compounds [2,3]. Our previous studies have found that UHP can extend the shelf life of shrimp, scallops, oysters, and grass carp fillets and enhance their physical properties such as textural characteristics, color, water, and pH [4]. Proteins are the essential nutrients in aquatic products, and UHP treatment promotes different degrees of dissociation, unfolding denaturation, and aggregation of protein structures, leading to protein degradation and oxidation [5,6]. Notably, numerous studies have reported that, compared with the combined treatment of UHP and the two-step heating method, the quality characteristics of surimi products, such as structural properties, gelation attributes, and water-holding capacity, were enhanced through a single UHP treatment [7,8]. Consequently, the overall quality of surimi products can be effectively ensured.

In our preliminary studies, we investigated the effects of UHP treatment (200–400 MPa with holding times of 10–30 min) on the quality of fish balls. The highest sensory scores and colors were obtained in the 300 MPa 20 min UHP treatment, and the 400 MPa 10 min UHP treatment significantly promoted the migration of free water to bound water in the fish balls. The microstructure became denser, which enhanced the water retention capacity of the fish balls. Despite existing research on the effects of UHP on aquatic products, studies on the mechanisms of quality stabilization in fish balls during F-T cycles remain insufficient. Current investigations primarily focus on the direct impact of UHP on microbial inactivation, whereas systematic studies addressing its role in mitigating F-T-induced deterioration are limited. The effect of UHP treatment on the F-T stability of the fish balls was further investigated by examining the water-holding capacity, water distribution, mass properties, gel properties, microstructure, and oxidation of proteins and lipids during the F-T cycles. This study establishes a scientific foundation for prolonging the shelf life of food products and minimizing food waste.

## 2. Materials and Methods

### 2.1. Preparation and Freeze–Thaw Cycle of Sample

Frozen golden pomfret surimi (*Trachinotus ovatus*) was purchased from a local marine market (Changchun, China). Surimi was transported to the laboratory within 1 h in an insulated container with ice packs to maintain optimal temperature conditions. The preparation process for fish balls followed the method outlined by Jiang et al. with slight modifications [9]. The surimi was ground uniformly using a meat grinder at 500 rpm for 5 min. Surimi samples were washed separately with pre-cooled distilled water and 0.5% NaCl. Subsequently, additional ingredients, including 4% sucrose, 4% sorbitol, and 2% salt, were added to the mixture and mixed for 15 min. Next, 10% hydroxypropyl starch and 15% gelatin were added for seasoning and beating, respectively, for 15 min. The moisture content of the surimi was adjusted to 80% using ice water. All excipients used to prepare the fish balls were of food grade and commercially available.

Surimi (12 g) was shaped into balls using spherical molds (diameter, 3 cm). Some fish balls (*n* = 30 per group) were randomly selected for heating using a two-step procedure (heated at 40 °C for 30 min, followed by 90 °C for 20 min in a water bath) as a control group (Con). The other samples underwent UHP treatment (HPP.L2-600/1; Tianjin Huatai Senmiao, Tianjin, China). The UHP treatment parameters were 300 MPa for 20 min (UHP_300-20_) and 400 MPa for 10 min (UHP_400-10_). The fish balls were put into plastic bags, frozen at −20 °C for 24 h, and then thawed at 4 °C for 12 h as an F-T cycle. Measurements of relevant parameters were performed after each of the five F-T cycles.

### 2.2. Water Holding Capacity (WHC)

#### 2.2.1. Thawing Loss

The total weight of the sample, juice, and bag was accurately recorded (M1). The bag was cut open, and the juices on the inside and the exterior of the fish balls were blotted dry using filter paper. Then, the weight of the sample and bag was recorded after drying the surface (M2). Finally, the polyvinylchloride bag was weighed (M3). Using the following formula:

(1) $$Thawing\;loss\left(\%\right)=\frac{M_{1}-M_{2}}{M_{1}-M_{3}}\times 100\%$$

#### 2.2.2. Centrifugal Water Loss

Following a previous protocol [10], fish ball samples (W_1_) were surrounded by three sheets of filter paper and subjected to centrifugation at 5000× *g* for 15 min at 4 °C (Allegra X-30R; Beckman Coulter, Brea, CA, USA). After centrifugation, the samples were removed and reweighed (W_2_). The results were obtained using Equation (2):

(2) $$Centrifugal\;water\;loss\left(\%\right)=\frac{W_{1}-W_{2}}{W_{1}}\times 100\%$$

#### 2.2.3. Cooking Loss

The mass of the minced fish used to prepare the fish balls was recorded as m_1_. The prepared fish balls were placed in a 75 °C-water bath for 25 min; following which, their weight was recorded as m_2_. We calculated the cooking loss as follows:

(3) $$Cooking\;loss\left(\%\right)=\frac{m_{1}-m_{2}}{m_{1}}\times 100\%$$

### 2.3. Low-Field Nuclear Magnetic Resonance (LF-NMR)

Water distribution was tested using LF-NMR (Suzhou Niumag Analytical Instrument Co., Suzhou, China), according to the method described by Ding et al. [11]. Fish balls were wrapped individually and placed in test tubes. The conditions were as follows: tube diameter, 40 mm; TE, 0.160 ms; NECH:12500; NS, 8.

### 2.4. Determination of Texture

Texture was assessed as described by Liu et al. [12]. A texture analyzer (TA.XT.Plus, Stable Microsystems, Godalming, UK) was used in the standard TPA mode. The test parameters were as follows: pre-test speed, 3 mm/s; test speed, 3 mm/s; post-test speed, 5 mm/s; trigger force, 5 g; probe model, P/5. The compression level was set at 50%, and the probe was pressed twice at 5 s intervals.

### 2.5. Determination of Gel Strength

The gel strength of the thawed fish ball samples was measured using a texture analyzer (TA.XT.Plus, Stable Micro Systems, Godalming, UK) as described by Luo et al. [13]. The analysis parameters were as follows: probe type, P/0.5S spherical probe; test speed, 1 mm/s; pressed down twice at 5 s intervals; trigger force, 5 g; and 50% compression ratio.

### 2.6. Color Evaluation

L* (lightness), a* (redness/greenness), and b* (yellowness/blueness) values were recorded using a colorimeter (CM 5; Konica Minolta, Singapore), and the whiteness (W value) was calculated using Equation (4), as follows:

(4) $$W=100-{\left[{\left(100-L*\right)}^{2}+{a*}^{2}+{b*}^{2}\right]}^{\frac{1}{2}}$$

### 2.7. Chemical Interaction

Chemical interaction was determined according to the method described by Mi et al. [14]. The fish balls were treated with a solution consisting of 10 mL of SA (0.05 mol/L NaCl), SB (0.6 mol/L NaCl), SC (0.6 mol/L NaCl+ 1.5 mol/L urea), SD (0.6 mol/L NaCl + 8 mol/L urea), and SE (0.6 mol/L NaCl + 8 mol/L urea + 0.05 mol/L β-mercaptoethanol). This treatment aimed to disrupt specific molecular interactions. After homogenization, the samples were centrifuged at 10,000× *g* for 10 min. The protein concentration in the supernatant was determined using Coomassie Brilliant Blue (CBB) assay. The differences between SA–SB, SB–SC, SC–SD, and SD–SE were used to quantify ionic bonds, hydrogen bonds, hydrophobic interactions, and disulfide bonds, respectively.

### 2.8. Morphology of Ice Crystals

The balls were cut into small pieces and dehydrated. The wax-embedded samples were sectioned longitudinally into 4 μm slices, and the paraffin was removed. The sections were stained with hematoxylin and eosin for 5 min, dehydrated using graded alcohol solutions, sealed with neutral resin, and observed under a light microscope.

### 2.9. Scanning Electron Microscopy (SEM)

Thawed fish ball samples were cut into 2–3 mm cubes and immediately fixed in 2.5% glutaraldehyde solution at 4 °C for 12 h. Following three rinses in 0.2 mol/L phosphate buffer (pH 7.2), the samples were dehydrated using a gradient of ethanol, starting with 50%, with increments of 10% up to 100%. The samples were subsequently freeze-dried, sectioned, and sprayed with gold prior to observation under a scanning electron microscope.

### 2.10. Myofibrillar Protein (MP) Extraction

Chopped fish balls (2 g) were mixed with 15 mL of 20 mmol/L Tris-HCl buffer (pH 7.5) and homogenized. The supernatant obtained after centrifugation was designated as the MP extract. The protein concentration in the MP solution was determined using the biuret method. The solution concentration was adjusted to 4 mg/mL for further analysis.

### 2.11. Protein Oxidation

The protein carbonyl content was determined using the 2,4-dinitrophenylhydrazine (DNPH) method as described by Oliver et al. [15], with slight modifications. Briefly, 1 mL of MP solution was mixed with 1 mL of 10 mM DNPH solution. The mixture was incubated in the dark for 1 h at 25 °C. Then, 1 mL of 20% trichloroacetic acid was added, and centrifuged for 5 min to collect the precipitate (10,000× *g*, 4 °C). The sediment was rinsed three times with a 1:1 (*v*/*v*) mixture of ethyl acetate and ethanol and dissolved in 3 mL of a 6 mol/L guanidine hydrochloride solution. The absorbance was recorded at 370 nm, and the carbonyl content was calculated as nanomoles of carbonyls per mg of protein. The levels of free sulfhydryl (SH) groups and disulfide bonds (S–S) were evaluated using the method described by Zeng [5].

### 2.12. Peroxide Value (POV)

POV was measured according to the method described by Xu [16]. Lipids were extracted using a previously established method with some modifications [17]. A 10 g portion of minced fish balls was homogenized with 150 mL of a chloroform–methanol mixture (2:1, *v*/*v*) for 3 min. The mixture was filtered and washed twice with 25 mL of chloroform. The filtrate (37.5 mL) of the potassium chloride solution (0.88 g/100 mL) was transferred to a dispensing funnel. The samples were allowed to stand overnight to stratify and collect the underlying organic components, and were concentrated by rotary evaporation (RE-52AA, Shanghai Yarong Biochemical Instrument Factory, Shanghai, China) at 40 °C. Nitrogen blowing till constant weight (OA-HEAT, Organomation Company, Berlin, MA, USA) was used to obtain the pure oil product.

### 2.13. Thiobarbituric Acid Reactive Substances (TBARSs)

Lipid oxidation in the fish ball samples was evaluated by measuring TBARSs following the method described by Zhou et al., with minor modifications [18]. First, a standard curve was constructed using different concentrations of malondialdehyde. To 0.2 g of the chopped sample, 1 mL of color development solution (0.375% thiobarbituric acid, 15% trichloroacetic acid, and 0.25 mol/L HCl) was added, followed by homogenization for 30 s. The samples were heated in boiling water for 10 min. After cooling, centrifugation was performed at 4500× *g* for 5 min. The supernatants were collected, and the absorbance was measured at 532 nm (FLUOstar Omega, BMG LABTECH, Ortenberg, Germany). The TBARS value was expressed as milligrams of MDA equivalents per kilogram of muscle.

### 2.14. Statistical Analysis

Experimental results were presented as mean ± standard deviation (*n* = 3). The statistical analyses were performed using GraphPad Prism 8.0 and Origin 2022. Two-way analysis of variance (ANOVA) was used to determine significant differences (*p* < 0.05) using SPSS 20.0.

## 3. Results and Discussion

### 3.1. Changes in WHC of Fish Balls

**Figure 1:**
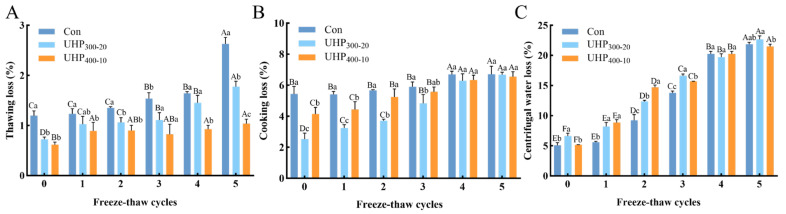
Effects of UHP treatment on water retention capacity during freeze–thaw cycles of fish ball (**A**) Thawing loss, (**B**), Cooking loss (**C**) Centrifugal water loss. Note: Differences in lowercase letters represent significant differences between treatment groups (*p* < 0.05). Differences in capital letters represent significant relationships between the same treatment groups during freeze–thaw cycles (*p* < 0.05).

WHC, a key quality attribute of aquatic products, reflects the water retention ability of the muscle tissue. To evaluate the WHC of fish balls under F-T conditions, key indicators, including thawing loss, centrifugal water loss, and cooking loss, were measured. As shown in Figure 1, an increase in the number of F-T cycles led to a significant increase (*p* < 0.05) in the thawing, centrifugation, and cooking loss rates for all groups. However, no significant differences were found in the cooking loss rates among the groups (*p* > 0.05). The thawing losses of the Con, UHP_300-20_, and UHP_400-10_ groups increased by 1.19, 1.37, and 0.68 times, respectively, and the centrifugal water loss rate was enhanced by 3.31, 2.43, and 3.16 times, respectively. The results indicated that a tighter gel network was formed in the fish balls upon UHP treatment, which made the structure less prone to loosening during the F-T cycles, reduced water loss, and improved the juiciness and texture. When the treatment condition was 400 MPa for 10 min, the thawing losses were reduced across all the F-T cycles. The reduction in water loss may be attributed to the pressure-induced orderly aggregation of proteins during UHP_400-10_ treatment, which enhances water binding and reinforces the gel network, thereby reducing thawing loss [19]. In addition, heating and different conditions of UHP treatment disrupt protein–water interactions to different degrees, and the ability to trap water molecules in the gel network varies [20]. Further research is required to elucidate the fundamental mechanisms underlying the water loss.

### 3.2. Changes in Water State and Water Distribution in Fish Ball

**Figure 2:**
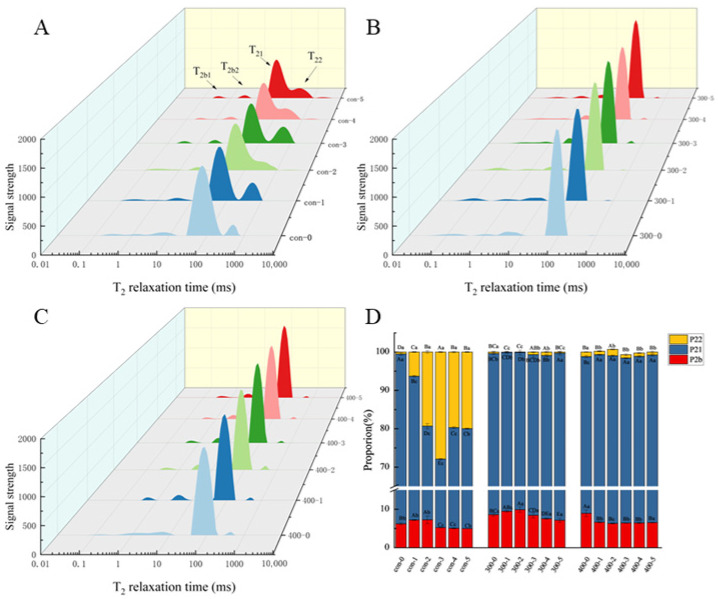
Effects of UHP treatment on T_2_ relaxation (spin-spin relaxation) during freeze–thaw cycles of fish ball (**A**) Con group (**B**) UHP_300-20_ (**C**) UHP_400-10_ (**D**) peak area ratio. Note: Differences in lowercase letters represent significant differences between treatment groups (*p* < 0.05). Differences in capital letters represent significant relationships between the same treatment groups during freeze–thaw cycles (*p* < 0.05).

Analysis of the spin–spin relaxation time (T_2_) using LF-NMR clarified the state and distribution of water in the fish balls during temperature fluctuations. The different types of water were characterized by three proton signals: T_2b1_ (0.01–1 ms) and T_2b2_ (1–10 ms) are characteristic peaks of bound water, T_21_ (10–200 ms) and T_22_ (>200 ms) correspond to fixed and free water, respectively [21]. Figure 2A–C show the relaxation spectra of the three groups under F-T cycles. The results demonstrate that the relaxation time (T_2_) of the groups remained unchanged irrespective of the number of F-T cycles. At the same time, UHP treatment led to significantly shorter T_2_ relaxation times than those of the samples that underwent heat treatment (*p* < 0.05). UHP treatment affects intermolecular interaction forces by altering the MP structure, resulting in a strong association between the protein network and water and attenuated mobility [22]. Furthermore, the freezing point of water is lowered in high-pressure environments, resulting in smaller ice crystals forming as water begins to freeze at lower temperatures. The reduction in the size of ice crystals is less destructive to the cellular structure of tissues, potentially reducing cell wall rupture and water loss throughout the F-T cycles. The T_2b_ bound water exhibited double (0.1–1 ms and 1–10 ms) or even triple peaks upon both heat and UHP treatment. The stretching of molecular chains in the pasted starch may account for the phenomenon, leading to a reduction in the strength of the abso rption peaks and the dispersion of bound water into multiple peaks [23]. As shown in Figure 2D, P_2b_, P_21_, and P_22_ represent the proportions of the corresponding peak areas. The UHP treatments, especially UHP_300-20_, facilitated the mobility of water molecules towards bound water and enhanced the proportion of bound water, limiting water migration during the F-T cycles. The combined water is less likely to form ice crystals during freezing, decreasing the number and size of ice crystals and improving the frost resistance of the fish balls, thus improving their F-T stability. Overall, both UHP treatment conditions increased the water retention capacity of the fish balls and limited the flow of water during the F-T cycles.

### 3.3. Changes in Physicochemical Properties and Color in Fish Balls

Water loss can change essential quality attributes such as color and texture. Physical characteristics are the key metrics for assessing the quality of fish mince products. UHP enhances the interaction between the matrix components and alters the water distribution, thus affecting the textural properties of the fish balls. Our previous research has shown that pressure above 200 MPa can significantly affect the textural characteristics of grass carp fillets, which could be associated with protein denaturation and an oxidation-driven conformation [24]. As shown in Table 1 after five F-T cycles, the hardness, elasticity, chewiness, and adhesiveness of the Con group fish balls decreased by 32.55%, 34.74%, 31.84%, and 6.40%, respectively, and the various texture characteristics of the UHP_300-20_ group fish balls decreased by 56.80%, 3.32%, 39.21%, and 12.87%, respectively, while those of the UHP_400-10_ group decreased by 67.17%, 4.84%, 51.72%, and 21.78%, respectively. It was demonstrated that the UHP-treated fish balls retained their hardness, chewiness, and bonding poorly, but their elasticity was better retained during the F-T cycles. The greater decline rates observed in the UHP-treated groups indicate that the UHP treatment resulted in poorer textural stability. UHP itself enhances non-covalent bonding during protein gelation and the formation of fragile and unstable gels [25]. Consequently, proteins are more prone to dehydration and condensation after F-T cycles and endogenous enzyme hydrolysis.

However, UHP-induced incomplete inactivation of microorganisms leads to poor texture, and F-T cycles accelerate microbial growth [26,27]. In addition, UHP-treated fish balls exhibited enhanced viscoelasticity after five cycles. More water molecules are trapped in the gel network by the UHP treatment, thus increasing the viscoelasticity of the gel. Compared to heat treatment alone, UHP treatment enhanced the energy storage and loss moduli of the MP gels, which gradually developed a dynamically balanced bonding state with increasing F-T cycles, resulting in optimal mechanical properties [28].

**Table 1:** Texture characteristics of fish balls in each treatment group during freeze–thaw cycles.

Freeze–Thaw Cycles	Group	Hardness(kg)	Springiness	Chewiness	Adhesive Properties
0	Con	345.194 ± 18.390 ^Aa^	0.734 ± 0.115 ^Ab^	64.086 ± 3.062 ^Ab^	0.504 ± 0.050 ^Ac^
	UHP_300-20_	172.501 ± 10.709 ^Ac^	0.963 ± 0.018 ^Aa^	39.988 ± 4.927 ^Ac^	0.575 ± 0.042 ^Ab^
	UHP_400-10_	262.143 ± 3.592 ^Ab^	0.991 ± 0.012 ^Aa^	73.826 ± 3.564 ^Aa^	0.684 ± 0.022 ^Aa^
1	Con	319.825 ± 12.939 ^Ba^	0.707 ± 0.036 ^Ab^	63.775 ± 2.585 ^Aa^	0.486 ± 0.114 ^Aa^
	UHP_300-20_	167.544 ± 3.228 ^Ac^	0.927 ± 0.037 ^Ba^	35.094 ± 2.362 ^Bb^	0.575 ± 0.060 ^Aa^
	UHP_400-10_	199.236 ± 7.002 ^Bb^	0.954 ± 0.025 ^Ba^	60.905 ± 3.172 ^Ba^	0.596 ± 0.074 ^Ba^
2	Con	289.863 ± 14.156 ^Ca^	0.772 ± 0.093 ^Ab^	63.449 ± 2.125 ^Aa^	0.540 ± 0.050 ^Aa^
	UHP_300-20_	151.126 ± 9.578 ^Bb^	0.842 ± 0.025 ^Cb^	30.852 ± 2.979 ^Bc^	0.538 ± 0.023 ^ABa^
	UHP_400-10_	78.392 ± 23.434 ^Dc^	0.927 ± 0.033 ^BCa^	50.635 ± 1.937 ^Cb^	0.541 ± 0.027 ^BCDa^
3	Con	249.506 ± 6.427 ^DEa^	0.750 ± 0.135 ^Ab^	54.499 ± 2.777 ^Ba^	0.451 ± 0.057 ^ABb^
	UHP_300-20_	146.877 ± 2.540 ^Bb^	0.740 ± 0.012 ^Db^	32.291 ± 3.035 ^Bc^	0.533 ± 0.026 ^ABa^
	UHP_400-10_	96.603 ± 6.965 ^Cc^	0.910 ± 0.023 ^Ca^	45.633 ± 1.838 ^Db^	0.580 ± 0.058 ^BCa^
4	Con	265.788 ± 19.928 ^Da^	0.752 ± 0.140 ^Ab^	47.231 ± 2.420 ^Ca^	0.454 ± 0.037 ^ABc^
	UHP_300-20_	148.689 ± 3.587 ^Bb^	0.740 ± 0.003 ^Db^	33.507 ± 4.121 ^Bb^	0.545 ± 0.021 ^ABa^
	UHP_400-10_	84.858 ± 4.682 ^CDc^	0.906 ± 0.039 ^Ca^	46.431 ± 2.869 ^Da^	0.495 ± 0.038 ^Db^
5	Con	232.834 ± 17.367 ^Ea^	0.479 ± 0.092 ^Bb^	43.678 ± 1.998 ^Da^	0.377 ± 0.112 ^Bb^
	UHP_300-20_	74.509 ± 7.645 ^Cc^	0.931 ± 0.023 ^Ba^	31.188 ± 2.757 ^Bc^	0.501 ± 0.039 ^Ba^
	UHP_400-10_	86.056 ± 16.466 ^CDb^	0.943 ± 0.012 ^Ba^	35.637 ± 1.873 ^Eb^	0.535 ± 0.031 ^CDa^

Differences in lowercase letters represent significant differences between treatment groups (*p* < 0.05). Differences in capital letters represent significant relationships between the same treatment groups during freeze–thaw cycles (*p* < 0.05).

**Figure 3:**
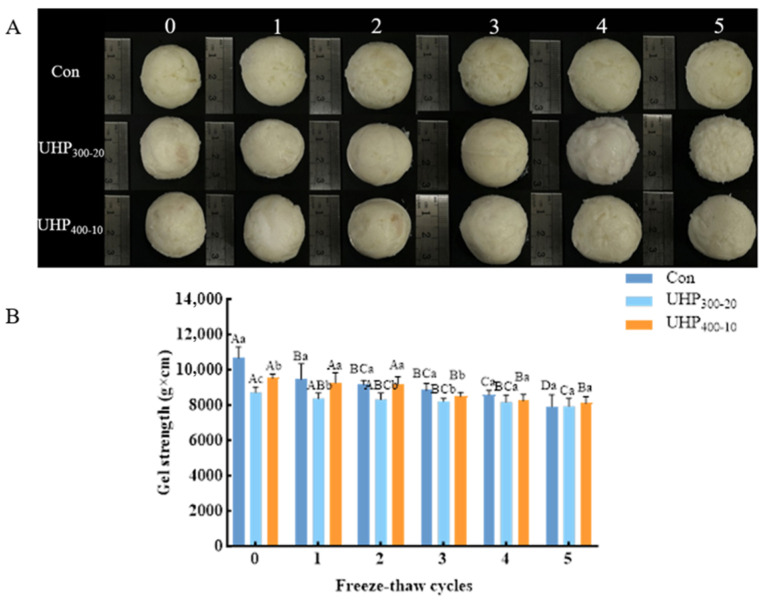
The appearance (**A**) and gel strength (**B**) of fish balls in each treatment group during freeze–thaw cycles. Note: Differences in lowercase letters represent significant differences between treatment groups (*p* < 0.05). Differences in capital letters represent significant relationships between the same treatment groups during freeze–thaw cycles (*p* < 0.05).

High-quality fish balls often require high whiteness, which affects consumer acceptability. Table 2 shows the chromaticity changes in the three groups of fish balls after the F-T cycles. Compared to unfrozen fish balls, UHP treatment resulted in high brightness (L*) and whiteness (W), which increased significantly with increasing pressure. UHP treatment may result in the degradation of certain pigments (e.g., myoglobin), reducing the depth of color in the sample and resulting in a relative increase in whiteness [29]. After five F-T cycles, the L* and W values significantly increased in the Con and UHP_300-20_ groups (*p* < 0.05), while the UHP-treated group remained significantly superior to the Con group (*p* < 0.05). The increased gloss of UHP_300-20_-treated fish balls after the F-T cycles indicated a smoother surface structure and heightened ability to reflect light. This is shown in Figure 3A, wherein the color and condition of the fish balls are presented intuitively. This outcome was contrary to that of Li et al., who found that the whiteness of mirror carp fish pellets was significantly reduced after seven F-T cycles [30], perhaps because of a difference in the type of gelatin in the fish balls, or the different ingredients and fish species used to prepare the fish balls. At lower temperatures, pressure has been reported to induce unfolding of myosin, which probably contributes to its “whitening” effect [31]. Concurrently, repeated F-T cycles resulted in severe surface cracking and shrinkage of the Con group, likely owing to the formation of large ice crystals and mechanical damage to the protein matrix. In contrast, the surfaces of the UHP_300-20_ and UHP_400-10_ fish balls remained smooth and intact, exhibiting minimal cracking. The preservation of structural integrity is associated with the ability of UHP to reduce ice crystal size and enhance water redistribution, thereby alleviating mechanical stress during the freezing process.

### 3.4. Changes in Gel Strength in Fish Balls

Gel strength was used to assess the firmness of the gel structure in the surimi products. The greater the breaking distance and force of a gel, the higher its strength and quality. As shown in Figure 3B, with an increase in the number of F-T cycles, the gel strength of fish balls in all groups significantly diminished (*p* < 0.05), mirroring the same trend as the hardness of the fish balls. The combined effect of ice crystals, internal enzymes, and oxygen after 5 F-T cycles at −20 °C inhibited the cross-linking of amino acid side chains in surimi proteins, which caused a gel network characterized by low-intensity properties. Compared to the unfrozen and thawed fish balls, the gel strength in the control, UHP_300-20_, and UHP_400-10_ groups decreased by 25.84%, 9.10%, and 15.00%, respectively; therefore, UHP treatment delayed this weakening effect. Similar findings were reported in mirror carp proteins, where F-T cycles caused aggregation-induced loss of gelling properties, while sodium alginate glazing helped reduce strength loss in surimi [30]. Remarkably, there was no significant difference in the gel strength of the fish balls in each group after 5 F-T cycles (*p* > 0.05). The enhancement in gel strength was ascribed to the modification of the molecular arrangement upon UHP treatment, which instigated orderly alterations in the secondary and tertiary structures of the protein, thereby forming a dense gel network that exhibited resistance to damage caused by ice crystal growth during the F-T cycles. Concurrently, according to Table S1, UHP_400-10_ treatment significantly increased the ionic and hydrogen bonding contents in the fish balls (*p* < 0.05). In a UHP environment, voids inside a protein molecule are compressed, resulting in a reduction in the molecular size. This conformational change allows some of the otherwise weakly interacting groups in the molecule to come into close contact. When the number of cycles reached five, the number of ion bonds, hydrogen bonds, hydrophobic interactions, and disulfide bonds in the Con group and UHP_400-10_ group fish balls were significantly reduced (*p* < 0.05). Although there were no significant differences in the ionic bonds and hydrophobic interactions in the UHP_300-20_-treated group, they also showed a decreasing trend. In brief, UHP treatment can effectively preserve ionic bonds and reduce the exposure of fish balls to hydrophobic groups, thus improving their functional properties and gelation ability [32].

### 3.5. Changes in Ice Crystal Morphology and Microstructure in Fish Balls

**Figure 4:**
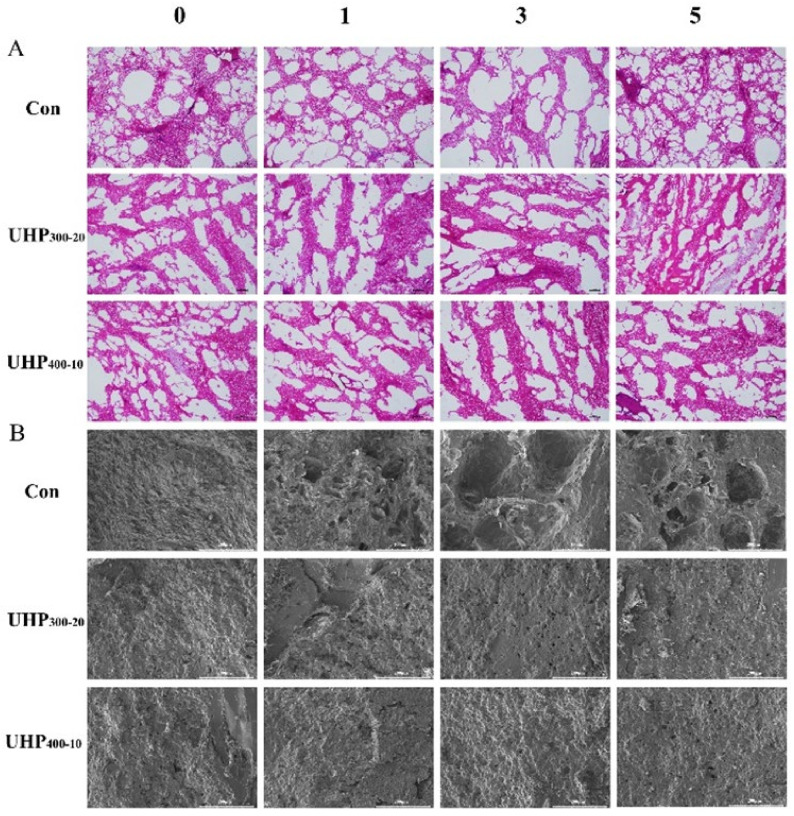
Effect of UHP treatment on the freeze–thaw cycling process of fish balls. (**A**) Optical microscope view of histology (×40) (**B**) Scanning electron microscope view of microstructure (×200).

To further confirm the impact and mechanism of UHP treatment on the gel properties of surimi during F-T cycling, changes in ice crystal morphology and myogenic fiber structure during temperature fluctuations were observed using H&E staining and SEM. The pink area corresponds to the muscle tissue and the white area corresponds to the traces left by the ice crystals (Figure 4A). Applying UHP without F-T cycles leads to structural changes in the sample muscle fibers, with a change in the geometry of the ice crystals from round to elongated stripes. All groups showed an increasing mean ice crystal size trend over the three F-T cycles. In addition, after five cycles, the muscle tissue was separated, and the ice crystals in each group became fragmented and irregular in shape, particularly in the Con group, where the changes were the most pronounced. The results indicated that the UHP treatment altered the distribution and geometry of ice crystals and maintained a slower growth rate during the freezing process. Under high-pressure conditions, some liquid water may be transformed into a non-crystalline state (e.g., glassy), which allows for the restricted movement of water molecules at low temperatures, thus reducing the freezing rate. Phase transitions in water molecules diminish the formation of large ice crystals during freezing [33].

Similar to the results observed upon H&E staining, the SEM images showed that after UHP treatment, the fish balls were flatter in cross-section, less porous, and had a compact and dense structure (Figure 4B). UHP treatment results in a more compact network structure, which can impede water penetration because water molecules have excellent resistance to diffusion through the gel structure. These results are consistent with previous conclusions regarding water distribution. In contrast, UHP treatment may promote the migration and interaction of different components (e.g., fats and sugars) in the fish balls. The redistribution and interaction of these components can further enhance the microstructural stability of the fish balls and refine their performance across F-T cycles. The improved microstructure also helps prevent oxidation reactions and enhances the product quality. The fish balls (control group) exposed to the two-step heating method exhibited an uneven cross-section and larger voids. Heat treatment facilitated starch gelatinization while concurrently leading to a reduction in α-helical structures and an increase in β-folding and random coil formations within the secondary structure of the proteins, which in turn led to the formation of dense but porous three-dimensional network gel structures [34]. Furthermore, with an increase in F-T events, the Con group structure exhibited distortion and experienced an increase in mechanical damage. Repeated penetration of ice crystals may lead to the formation of protein aggregates, thereby disrupting the network structure of the fish balls [35].

### 3.6. Changes in Protein and Lipid Oxidation in Fish Balls

#### 3.6.1. Protein Oxidation

**Figure 5:**
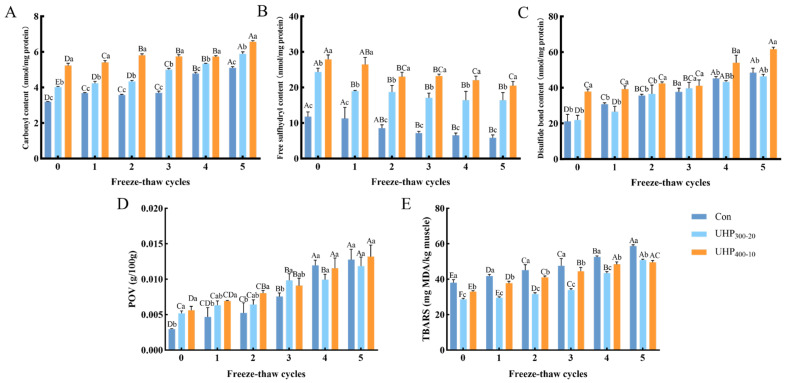
Effect of UHP treatment on the freeze–thaw cycling process of fish balls (**A**) Carbonyl content (**B**) Free sulfhydryl content (**C**) Disulfide bond content (**D**) POV (**E**) TBARS value. Note: Differences in lowercase letters represent significant differences between treatment groups (*p* < 0.05). Differences in capital letters represent significant relationships between the same treatment groups during freeze–thaw cycles (*p* < 0.05).

Carbonyl and sulfhydryl contents are key indicators of the extent of protein oxidation. Formation of carbonyl compounds affects the function and stability of proteins by influencing their structure. As shown in Figure 5A, UHP treatment promoted increased carbonylation in MPs compared to that in the Con group. After five F-T cycles, the carbonyl content increased by 60.07% in the control, 46.68% in the UHP_300-20_, and 25.54% in the UHP_400-10_ group compared with their respective initial values. This initial oxidative acceleration aligns with the results of previous studies, indicating that high-pressure conditions induce free radical generation, which promotes the oxidation of amino acid side chains and peptide backbones. UHP triggers protein unfolding, exposing free thiol groups in cysteine residues that can undergo further oxidation to form S–S bonds [36]. Figure 5B,C illustrate that the free SH content in the Con group decreased progressively from 11.79 nmol/mg to 5.83 nmol/mg with the rise in F-T cycles. In contrast, the free SH content of samples treated with UHP declined from 24.35 nmol/mg to 16.42 nmol/mg and from 27.89 nmol/mg to 20.49 nmol/mg, respectively. Inversely, with rising frequency of F-T cycles, the content of S–S bonds augmented gradually from 21.09 nmol/mg to 48.44 nmol/mg, from 21.98 nmol/mg to 46.34 nmol/mg, and from 37.91 nmol/mg to 61.57 nmol/mg for the three groups of samples, respectively. These results indicate that more buried sulfhydryl groups were exposed and activated under high pressure. F-T cycles are one of the reasons for the exposure of active sites, leading to increased sensitivity and oxidation degree of the S–S bond exchange.

Notably, despite having a higher initial oxidation level, the UHP_400-10_ group demonstrated a slower rate of carbonyl accumulation (25.54%) than the control group (60.07%). This seemingly paradoxical outcome can be explained by two synergistic mechanisms. First, UHP-induced protein aggregation and cross-linking, such as the formation of disulfide (S–S) bonds, result in a dense gel network that physically obstructs the diffusion of oxidative mediators, including oxygen and free radicals, into the protein matrix [37]. Second, UHP may enhance endogenous antioxidant capacity by modulating water–ice phase transitions, which helps mitigate ice-crystal-induced mechanical damage and subsequent oxidative stress [38].

#### 3.6.2. Lipid Oxidation

Lipid oxidation leads to the production of harmful compounds and unpleasant odors, which negatively impact product quality. The peroxide value can be used to characterize the degree of primary lipid oxidation in the fish balls. Similar to protein, UHP treatment initially significantly promoted lipid peroxidation (*p* < 0.05), as evidenced by higher POV in UHP-treated groups, which increased by 1.28–1.35 times compared with the Con group (Figure 5D). With increasing F-T cycles, fish balls treated with UHP exhibited an enhanced antioxidant capacity and a slower tendency to increase POVs, which may be attributed to peroxide or free radical quenching due to fatty acid oxidation in fish balls [39].

TBARS assay can characterize the content of malondialdehyde, the final product of lipid oxidation [40]. As shown in Figure 5E, after five F-T cycles, the TBARS values of the fish balls increased by 54.02%, 77.24%, and 50.27% in the Con, UHP_300-20,_ and UHP_400-10_ groups, respectively. However, the TBARS value of fish pellets in the Con group (58.67 mg MDA/kg muscle) was still significantly higher than the remaining two groups (50.78 and 49.50 mg MDA/kg muscle). This is consistent with the findings of Cartagena et al., who observed that at a pressure of 200 MPa for 6 min, albacore demonstrated a reduction in the increase in TBARS values associated with frozen storage compared to non-pretreated albacore after 12 months of frozen storage [41]. Lipid oxidation is thought to occur at the cell membrane level, and the recrystallization of ice crystals during F-T disrupts the physical organization of membrane lipids, leading to muscle cell damage, which releases lipoxygenases and catalysts to enhance lipid oxidation. In contrast, UHP treatment reduced ice crystal growth and inhibited lipase activity.

Despite the initial exacerbation of protein and lipid oxidation by UHP, its dual mechanism of inducing structural crosslinking and inhibiting ice crystal damage ultimately delayed the overall oxidation rate during the F-T process. As a technological tool, UHP can potentially delay protein and lipid oxidation.

### 3.7. Correlation Analysis

**Figure 6:**
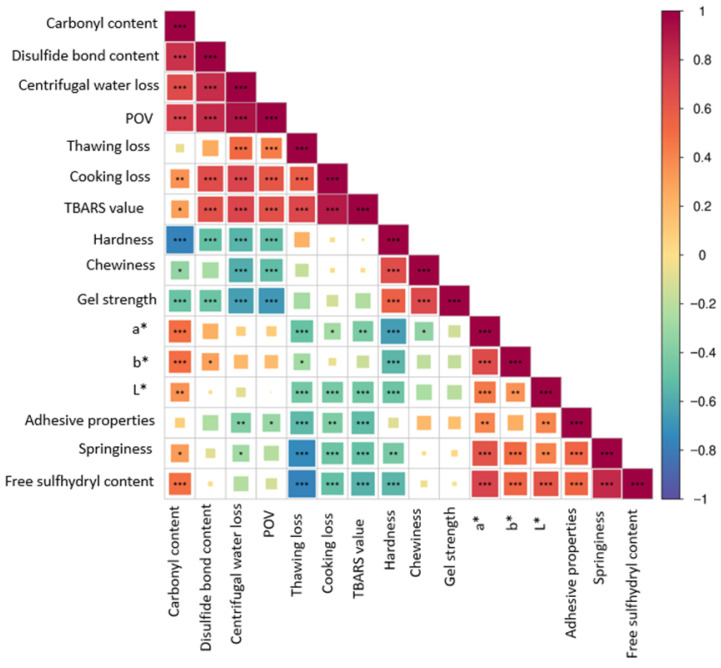
Pearson correlation heatmap of physicochemical and oxidative parameters in UHP-treated golden pomfret fish balls during freeze–thaw cycles. Colors represent the correlation coefficient (R), ranging from +1 (strong positive correlation, red) to −1 (strong negative correlation, blue). Asterisks denote significance levels: * *p* < 0.05, ** *p* < 0.01,*** *p* < 0.001.

Correlation analysis revealed intricate relationships between the quality parameters of golden pomfret fish balls subjected to UHP treatment during F-T cycles (Figure 6). The analysis revealed that a strong positive correlation between thawing loss and TBARS values (*p* < 0.001) indicated that water migration exacerbated lipid peroxidation via ice crystal-induced membrane damage. Hardness and gel strength were negatively correlated with the carbonyl content (*p* < 0.001), highlighting the effect of protein oxidation on the structure. Weaker POV-TBARS correlations in the UHP suggested decomposition of hydroperoxide into stable aldehydes/ketones, which reduced malondialdehyde accumulation. Negative correlations between free SH and TBARS (*p* < 0.001) implied that UHP-mediated S–S bonds stabilize proteins and suppress lipid oxidation.

These results have important practical significance for the surimi processing industry. UHP treatment demonstrated a unique ability to balance the initial oxidative acceleration with long-term stability through ice crystal modulation, protein network reinforcement, and regulation of oxidative pathways. This indicates that UHP can serve as a non-thermal preservation technology to improve the F-T stability, shelf life, and overall quality of surimi-based products. For the industry, adopting UHP technology would not only help reduce dependence on chemical additives, but will also minimize food waste caused by F-T damage, thereby promoting the development of clean-label frozen seafood products.

## 4. Conclusions

The results of this study indicate that UHP treatment of golden pomfret fish balls is an efficient approach for improving the fish ball quality during F-T cycles. UHP treatment resulted in a more uniform distribution of water in the fish balls, changed the water-binding state, and increased the proportion of bound water, thereby improving the WHC of the fish balls. In addition, after 0–5 F-T cycles, the physicochemical and gelation characteristics and color of the UHP-treated fish balls were superior to those prepared by the conventional two-step heating process. This improvement was attributed to the UHP treatment, which resulted in a uniform distribution of ice crystals, slower growth rates, preservation of muscle tissue integrity, and enhancement of the textural properties of the fish balls through a more regular arrangement of myogenic fibers. Although UHP initially increased protein and lipid oxidation, its dual action of promoting structural cross-links and suppressing ice crystal formation ultimately slowed oxidation during the F-T cycles. Correlation analysis revealed a positive correlation between moisture loss and lipid peroxidation, and a negative correlation between protein oxidation and structural characteristics. The UHP_400-10_ treatment demonstrated superior efficacy in reducing thawing loss and maintaining gel strength (10.84% decline), whereas UHP_300-20_ treatment excelled in minimizing cooking loss and preserved elasticity.

Overall, these findings emphasize the potential of UHP technology to address the quality challenges in surimi product processing. The long-term effects of UHP treatment on various surimi products should be explored in the future and their applicability to other food-processing environments should be investigated to further validate and extend their benefits.

## Figures and Tables

**Table 2:** Chromaticity of fish balls in each treatment group during freeze–thaw cycles.

Freeze–Thaw Cycles	Group	L*	a*	b*	W
0	Con	69.690 ± 0.909 ^Ec^	−3.132 ± 0.19 ^BCc^	1.568 ± 0.456 ^Bc^	69.485 ± 0.906 ^Ec^
	UHP_300-20_	78.153 ± 0.582 ^Db^	−2.478 ± 0.197 ^Bb^	2.687 ± 0.438 ^Bb^	77.846 ± 0.593 ^Db^
	UHP_400-10_	81.227 ± 0.902 ^Ca^	−2.033 ± 0.136 ^Aa^	3.598 ± 0.251 ^Aa^	80.775 ± 0.884 ^BCa^
1	Con	66.310 ± 0.762 ^Fb^	−2.823 ± 0.185 ^Ab^	1.970 ± 0.853 ^ABb^	66.126 ± 0.800 ^Fb^
	UHP_300-20_	83.417 ± 0.244 ^Aa^	−2.078 ± 0.035 ^Aa^	3.215 ± 0.188 ^ABa^	82.980 ± 0.263 ^Aa^
	UHP_400-10_	82.823 ± 0.594 ^Ba^	−2.203 ± 0.287 ^ABa^	3.045 ± 0.699 ^ABa^	82.403 ± 0.566 ^Ba^
2	Con	74.082 ± 0.359 ^Bb^	−3.322 ± 0.153 ^Cb^	2.605 ± 0.473 ^Ab^	73.736 ± 0.359 ^Bb^
	UHP_300-20_	82.837 ± 0.997 ^ABa^	−2.032 ± 0.06 ^Aa^	3.043 ± 0.784 ^ABab^	82.435 ± 0.960 ^Aa^
	UHP_400-10_	83.148 ± 0.523 ^Ba^	−2.162 ± 0.117 ^ABa^	3.653 ± 0.480 ^Aa^	80.643 ± 4.027 ^BCa^
3	Con	76.178 ± 0.842 ^Ac^	−2.898 ± 0.349 ^ABb^	1.877 ± 0.353 ^Bb^	75.926 ± 0.860 ^Ac^
	UHP_300-20_	79.932 ± 0.548 ^Cb^	−2.193 ± 0.191 ^Aa^	2.927 ± 0.768 ^ABab^	79.588 ± 0.515 ^Cb^
	UHP_400-10_	85.635 ± 0.500 ^Aa^	−2.582 ± 0.544 ^Bab^	3.453 ± 1.224 ^Aa^	84.951 ± 0.465 ^Aa^
4	Con	72.442 ± 0.179 ^Cc^	−3.302 ± 0.125 ^Cb^	2.247 ± 0.546 ^ABb^	72.149 ± 0.184 ^Cc^
	UHP_300-20_	82.200 ± 0.570 ^Ba^	−2.305 ± 0.183 ^ABa^	3.305 ± 1.109 ^ABa^	81.719 ± 0.502 ^Ba^
	UHP_400-10_	80.360 ± 0.337 ^Db^	−2.157 ± 0.509 ^ABa^	3.767 ± 0.605 ^Aa^	79.873 ± 0.300 ^Cb^
5	Con	71.568 ± 0.217 ^Dc^	−3.377 ± 0.116 ^Cb^	1.768 ± 0.439 ^Bb^	71.311 ± 0.227 ^Dc^
	UHP_300-20_	80.607 ± 0.214 ^Ca^	−2.108 ± 0.430 ^Aa^	3.822 ± 0.411 ^Aa^	80.115 ± 0.266 ^Ca^
	UHP_400-10_	79.308 ± 0.333 ^Eb^	−2.483 ± 0.559 ^ABa^	2.375 ± 0.760 ^Bb^	79.008 ± 0.356 ^Cb^

Differences in lowercase letters represent significant differences between treatment groups (*p* < 0.05). Differences in capital letters represent significant relationships between the same treatment groups during freeze–thaw cycles (*p* < 0.05).

## Data Availability

The original contributions presented in the study are included in the article/Supplementary Materials. Further inquiries can be directed to the corresponding author.

## Supplementary Materials

The following supporting information can be downloaded at: https://www.mdpi.com/article/10.3390/foods14193342/s1, Table S1: Chemical forces of fish balls in each treatment group during freeze–thaw.

## Abbreviations

The following abbreviations are used in this manuscript:

UHP	Ultra-high pressure
F-T	Freeze–thaw
LF-NMR	Low-field nuclear magnetic resonance
WHC	Water Holding Capacity
SEM	Scanning electron microscopy
MP	Myofibrillar protein
POV	Peroxide value
TBARS	Thiobarbituric acid-reactive substances
