# Supplementary material for: Mechanisms of Quality Preservation in Golden Pomfret Fish Balls Treated with Ultra-High Pressure During Freeze–Thaw Cycles
Source: Foods. 2025 Sep 26;14(19):3342. doi: 10.3390/foods14193342 (PMC12524334; doi:10.3390/foods14193342)
Supplement: Supplementary file 1 [file foods-14-03342-s001.zip › foods-3847508-supplementary.pdf]

Supplementary materials

Mechanisms of Quality Preservation in Golden Pomfret Fish Balls Treated with Ultra-High Pressure During Freeze-Thaw Cycles

Jiawen Liu[a,b], Xinyao Zeng[a,b], Jiaqi Zhao[a,b], Yunfeng Chi[a,b], Lin Xiu[a,b], Mingzhu Zheng[a,b], Huimin Liu[a,b,*]

[a]College of Food Science and Engineering, Jilin Agricultural University, Changchun, Jilin 130118, China

[b]National Engineering Laboratory for Wheat and Corn Deep Processing, Changchun, Jilin 130118, China

*Corresponding author

Dr. Huimin Liu

E-mail: liuhuimin@jlau.edu.cn

Jilin Agricultural University

Journal name：Food and Bioprocess Technology

Table S1 Chemical forces of fish balls in each treatment group during freeze-thaw cycles

Freeze-thaw cycles	Group	Ionic bond	Hydrogen bond	Hydrophobic interaction	S−S bond
0	Con	0.175 ± 0.004^{Ab}	0.132 ± 0.035^{Ab}	0.142 ± 0.052^{Aa}	0.345 ± 0.036^{Aa}
	UHP_{300-20}	0.187 ± 0.045^{Ab}	0.263 ± 0.018^{Aa}	0.140 ± 0.059^{Aa}	0.353 ± 0.035^{Aa}
	UHP_{400-10}	0.251 ± 0.023^{Aa}	0.282 ± 0.011^{Aa}	0.166 ± 0.016^{Aa}	0.360 ± 0.010^{Aa}
1	Con	0.146 ± 0.041^{ABa}	0.091 ± 0.034^{ABb}	0.084 ± 0.029^{Ba}	0.249 ± 0.043^{Ba}
	UHP_{300-20}	0.180 ± 0.019^{Aa}	0.238 ± 0.024^{Aa}	0.135 ± 0.066^{Aa}	0.250 ± 0.044^{Ba}
	UHP_{400-10}	0.171 ± 0.023^{ABa}	0.238 ± 0.043^{Aa}	0.153 ± 0.022^{Aa}	0.256 ± 0.022^{Ba}
2	Con	0.115 ± 0.029^{ABa}	0.069 ± 0.040^{ABb}	0.074 ± 0.047^{Bb}	0.242 ± 0.027^{Ba}
	UHP_{300-20}	0.168 ± 0.071^{Aa}	0.176 ± 0.034^{Ba}	0.104 ± 0.033^{Aa}	0.228 ± 0.035^{BCa}
	UHP_{400-10}	0.145 ± 0.059^{Ba}	0.232 ± 0.026^{Aa}	0.140 ± 0.023^{Aa}	0.242 ± 0.018^{Ba}
3	Con	0.089 ± 0.028^{BCa}	0.058 ± 0.026^{Bb}	0.056 ± 0.034^{Ba}	0.153 ± 0.022^{Ca}
	UHP_{300-20}	0.147 ± 0.092^{Aa}	0.116 ± 0.017^{Ca}	0.074 ± 0.103^{Aa}	0.180 ± 0.029^{CDa}
	UHP_{400-10}	0.137 ± 0.026^{Ba}	0.134 ± 0.028^{Ba}	0.089 ± 0.023^{Ba}	0.142 ± 0.010^{Ca}
4	Con	0.081 ± 0.063^{BCa}	0.038 ± 0.039^{Bb}	0.042 ± 0.018^{Ba}	0.145 ± 0.040^{Ca}
	UHP_{300-20}	0.140 ± 0.043^{Aa}	0.048 ± 0.033^{Db}	0.069 ± 0.055^{Aa}	0.164 ± 0.038^{CDa}
	UHP_{400-10}	0.124 ± 0.090^{Ba}	0.122 ± 0.023^{Ba}	0.077 ± 0.015^{Ba}	0.170 ± 0.016^{Ca}
5	Con	0.039 ± 0.009^{Ca}	0.060 ± 0.027^{Bab}	0.030 ± 0.002^{Ba}	0.128 ± 0.026^{Ca}
	UHP_{300-20}	0.112 ± 0.060^{Aa}	0.034 ± 0.020^{Db}	0.067 ± 0.075^{Aa}	0.146 ± 0.029^{Da}
	UHP_{400-10}	0.039 ± 0.010^{Ca}	0.116 ± 0.045^{Ba}	0.050 ± 0.051^{Ba}	0.150 ± 0.011^{Ca}

Note: The data represent the mean ± standard deviation of three repeated experiments. Differences in lowercase letters represent significant differences between treatment groups ($p < 0.05$). Differences in capital letters represent substantial relationships between the same treatment groups during freeze-thaw cycles ($p < 0.05$).
